# Supplementary figures and images for: Sorghum root epigenetic landscape during limiting phosphorus conditions
Source: Plant Direct. 2022 May 14;6(5):e393. doi: 10.1002/pld3.393 (PMC9107021; doi:10.1002/pld3.393)

**AP2/EREb (Hyper)**

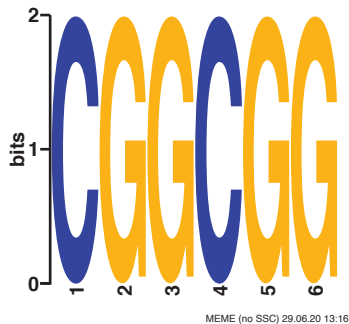

**C2H2 (Hyper)**

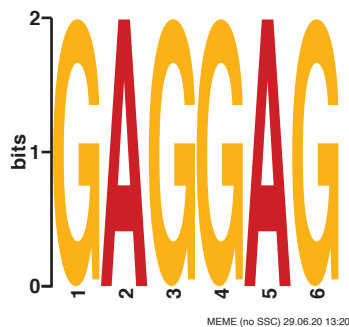

**TCP (Hyper)**

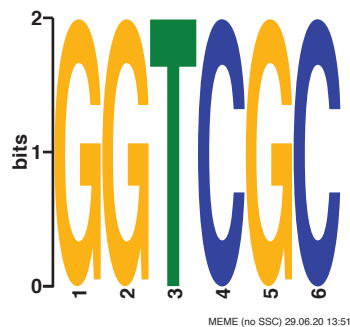

**bZIP (Hyper)**

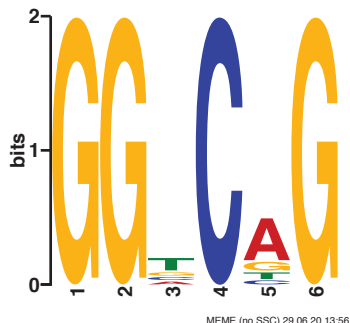

**bZIP (Hypo)**

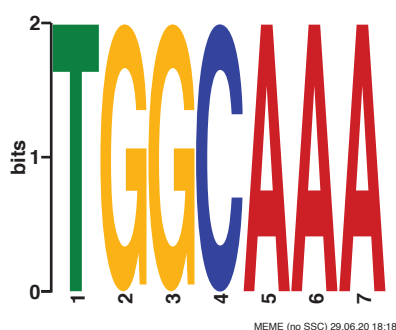

**NLP (Hypo)**

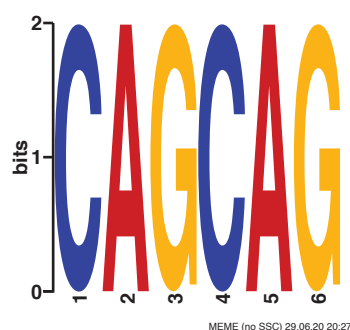

**BZR (Hyper)**

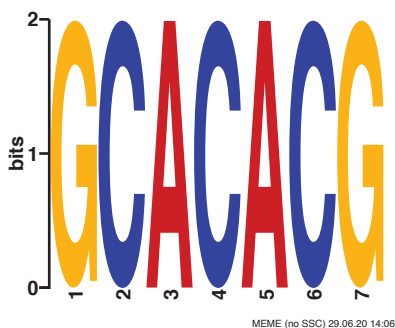

**NAC (Hypo)**

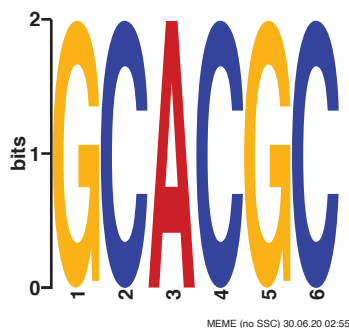

**Trihelix (Hypo)**

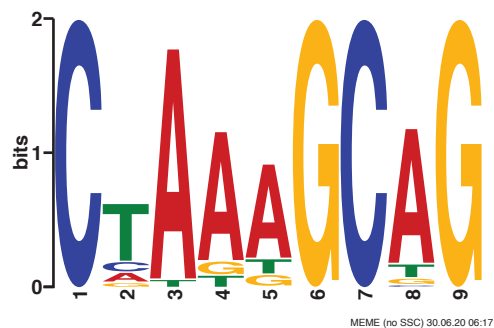

Supplement: Supplementary file 4 — Figure S1. DNA‐binding Motif Enrichment of CG Differentially Methylated Peaks. MEME analysis of the 100 bp surrounding differential CG 5‐methylcytosine modified regions in BTx623. All motifs were detected using the ANR method in the MEME suite program with minimum significance threshold of e < .01 or less. [file PLD3-6-e393-s004.pdf]

# AP2/EREB

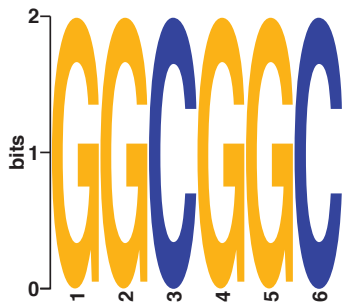

MEME (no SSC) 08.08.20 14:05

# LOB

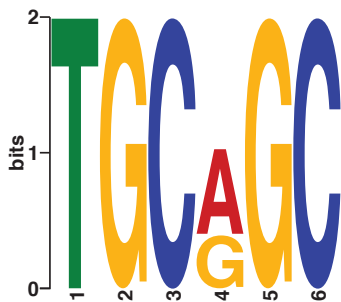

MEME (no SSC) 08.08.20 16:04

# WRKY

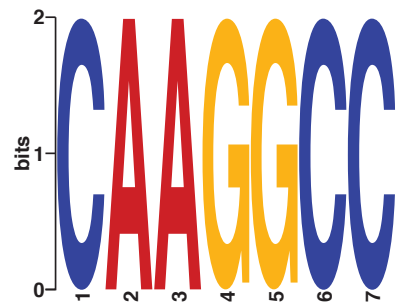

MEME (no SSC) 08.08.20 15:06

# TCP

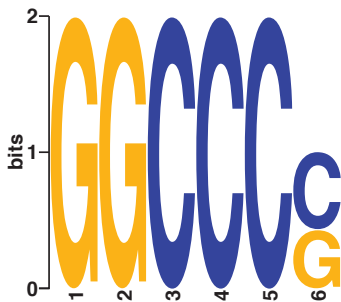

MEME (no SSC) 08.08.20 15:29

# EF2-like

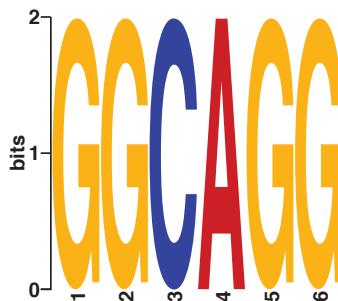

MEME (no SSC) 08.08.20 14:34

# NLP

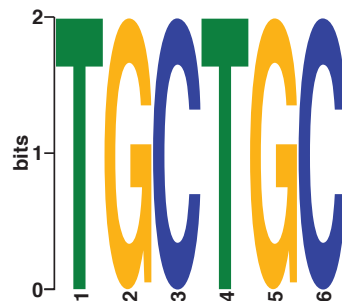

MEME (no SSC) 08.08.20 14:29

Supplement: Supplementary file 5 — Figure S2. DNA‐binding Motif Enrichment of CHG Differentially Methylated Peaks. MEME analysis of the 100 bp surrounding differential CHG 5‐methylcytosine modified regions in BTx623. All motifs were detected using the ANR method in the MEME suite program with minimum significance threshold of e < .01 or less. [file PLD3-6-e393-s010.pdf]

## WRKY (Hypo)

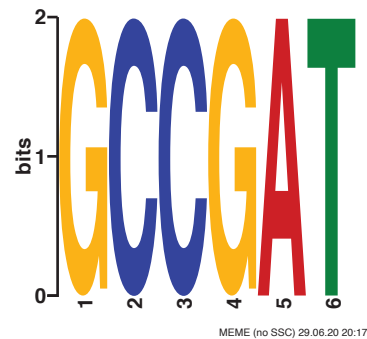

## NLP

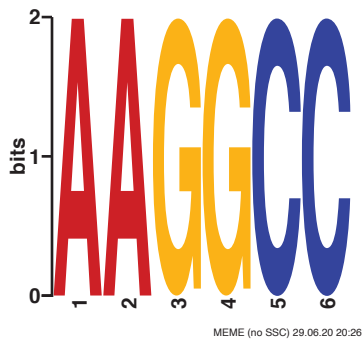

## AP2/EREB

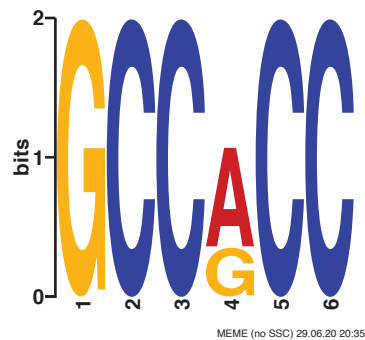

## TCP

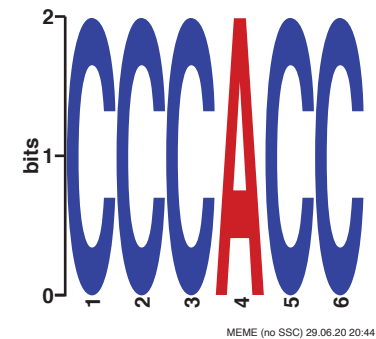

## NAC

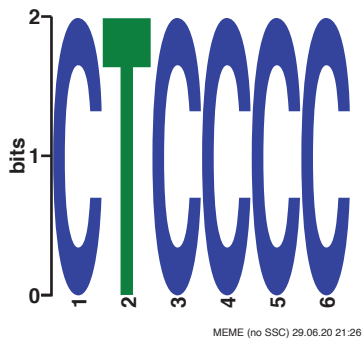

## bHLH (Hypo)

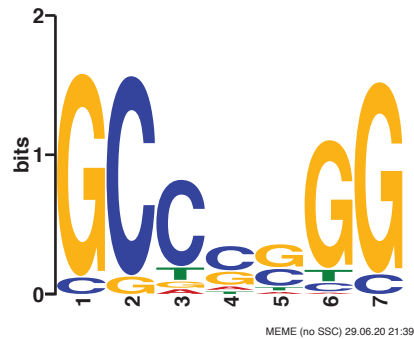

## Homeobox

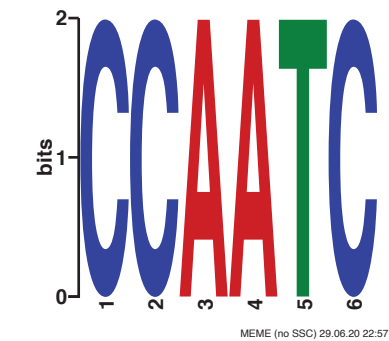

## ABI3 (Hypo)

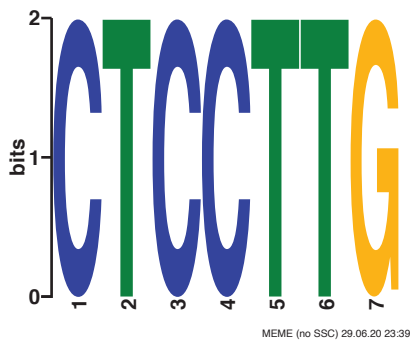

Supplement: Supplementary file 6 — Figure S3. DNA‐binding Motif Enrichment of CHH Differentially Methylated Peaks. MEME analysis of the 100 bp surrounding differential CHH 5‐methylcytosine modified regions in BTx623. All motifs were detected using the ANR method in the MEME suite program with minimum significance threshold of e < .01 or less. [file PLD3-6-e393-s001.pdf]

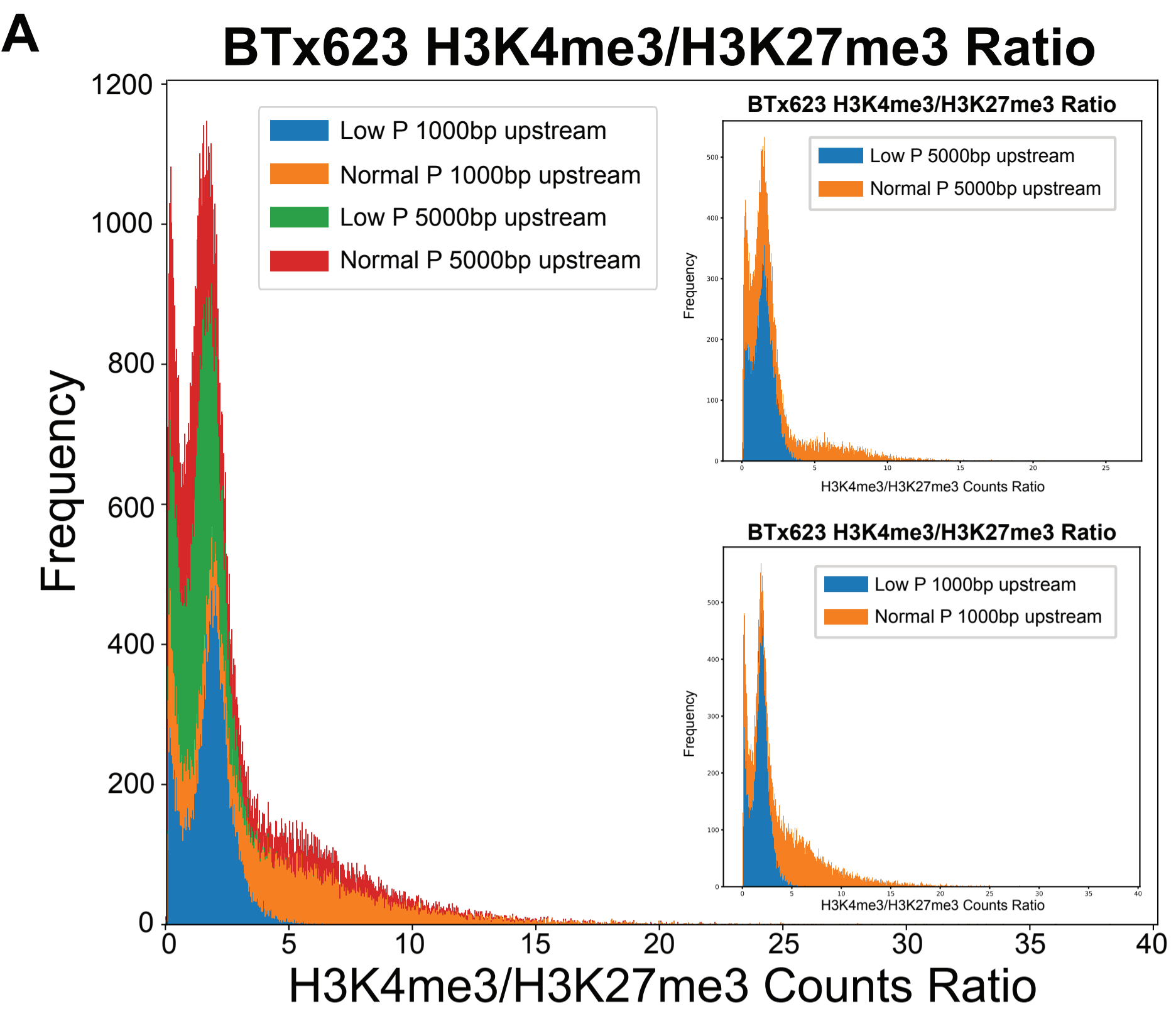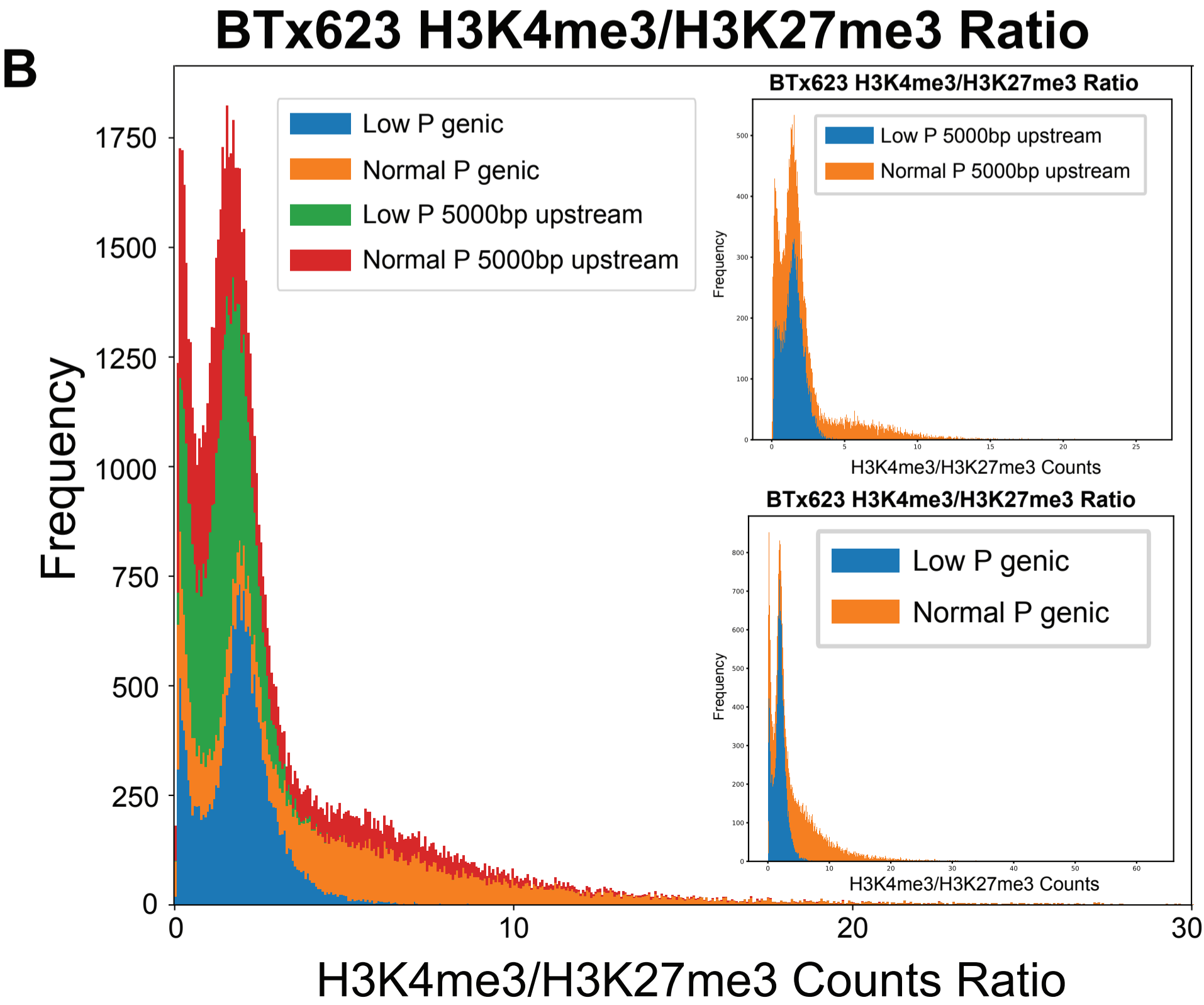

Supplement: Supplementary file 7 — Figure S4. K4/K27 Trimethylation Ratio Footprints Change from SP to LP. Frequency histograms comparing the global H3K4me3/H3K4me3 counts ratios that cover genic, and 1,000 bp upstream and 5,000 bp upstream (starting from TSS) regions in the BTx623 genome. Upon LP, the count ratios tend to coalesce to a lower value, shrinking back from the longer tails of larger H3K4me3/H3K27me3 ratios in SP conditions. Only regions where K4me3 and K27me3 counts were greater than zero in both SP and LP were included in this analysis. [file PLD3-6-e393-s003.pdf]

## SORBI\_3008G179900 O-acetylserine (thiol) Lyase

LP

SP

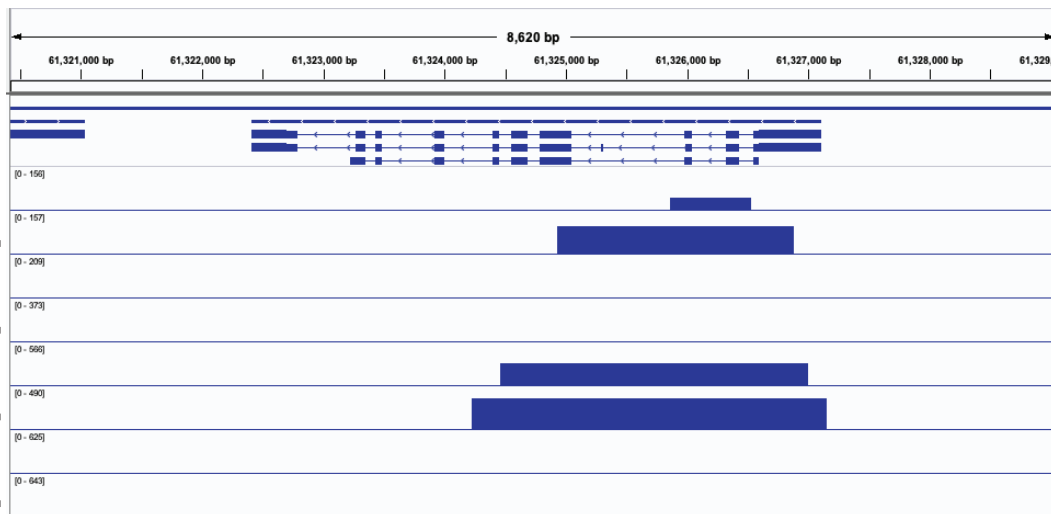

## SORBI\_3001G427300 Glycosyl Transferase

LP

SP

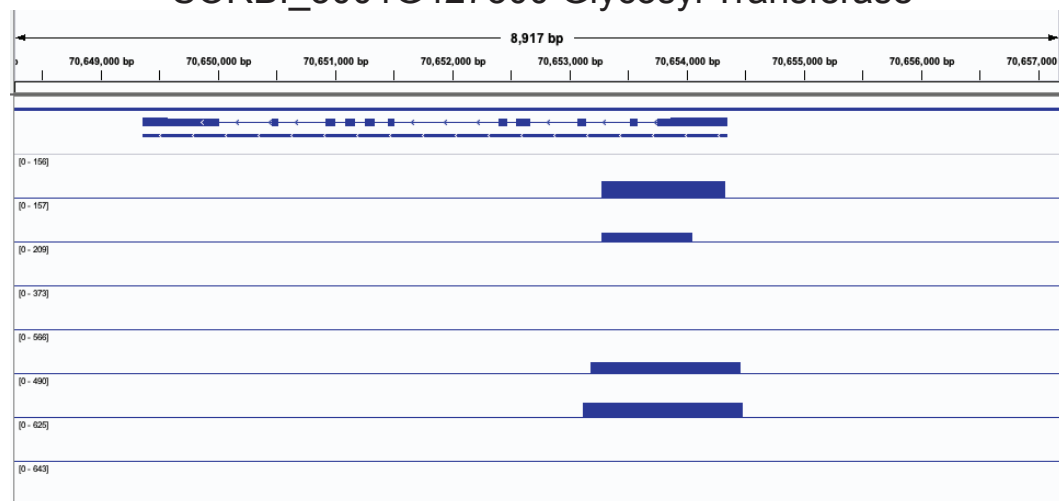

Supplement: Supplementary file 9 — Figure S6. Peak Occurrence of Selected Cysteine/Sulfur Metabolism Genes. Integrated Genome Viewer display of H3K4 and H3K27 trimethylation enriched peaks on the promoter/genic region of SORBI_3008G179900 and SORBI_001G427300 gene models. The display shows the two biological replicates for each histone mark during LP (top four panels) and SP (bottom four panels). Only the K4me3 trimethylation peaks are prominent for these genes and the peak region becomes smaller in the LP condition for both replicates. [file PLD3-6-e393-s007.pdf]
