# Supplementary material for: Sorghum root epigenetic landscape during limiting phosphorus conditions
Source: Plant Direct. 2022 May 14;6(5):e393. doi: 10.1002/pld3.393 (PMC9107021; doi:10.1002/pld3.393)
Supplement: Supplementary file 8 — Figure S5. RNA‐seq Expression Heatmaps of Selected Transcription Factor Family Genes. Gene expression across root regions for A) WKRY, B) NAC, C) bZIP, D) B3‐domain containing, and E) AP2 transcription factor family genes. GO enrichment of top‐expressed transcription factors in LP in the lateral root regions returns auxin‐activated signaling pathway (GO:0009734). The negatively expressed genes in LP in the apex region return response to chitin (GO:0010200) and abscisic acid‐activated (ABA) signaling pathway (GO:0009738) as well as the auxin‐activated signaling pathway (GO:0009734). Hierarchical clustering was performed with the built‐in heatmap.2 function in rStudio. [file PLD3-6-e393-s009.pdf]

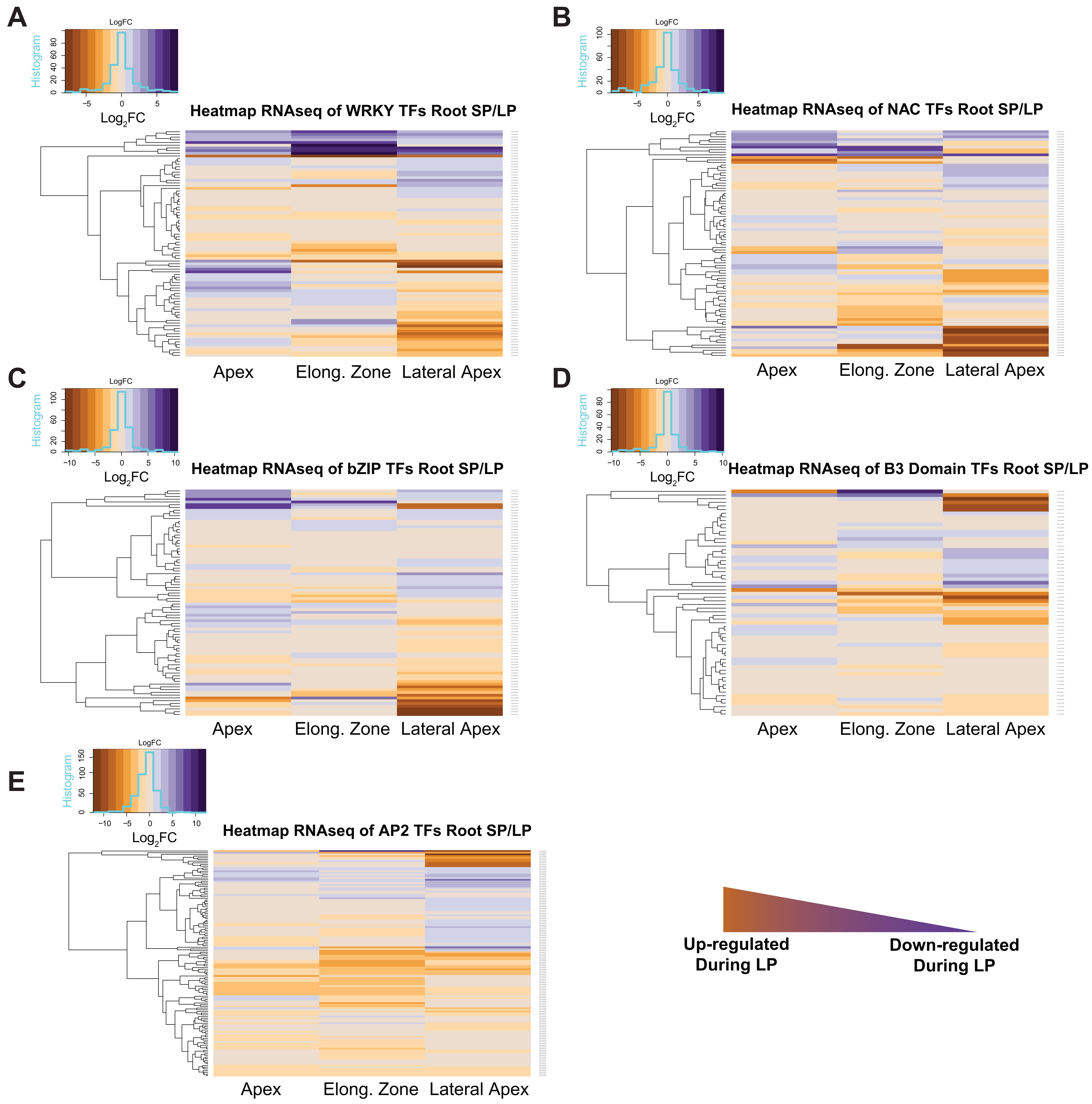

A
LogFC
Histogram
Log2FC
Heatmap RNAseq of WRKY TFs Root SP/LP
Apex
Elong. Zone
Lateral Apex
B
Heatmap RNAseq of NAC TFs Root SP/LP
C
Heatmap RNAseq of bZIP TFs Root SP/LP
D
Heatmap RNAseq of B3 Domain TFs Root SP/LP
E
Heatmap RNAseq of AP2 TFs Root SP/LP
Up-regulated During LP
Down-regulated During LP
