# Supplementary material for: Sorghum root epigenetic landscape during limiting phosphorus conditions
Source: Plant Direct. 2022 May 14;6(5):e393. doi: 10.1002/pld3.393 (PMC9107021; doi:10.1002/pld3.393)
Supplement: Supplementary file 10 — Figure S7. Model depicting DNA methylation and H3K4 and H3K27 trimethylation decreasing globally during growth under limiting phosphorus conditions. This shift in epigenetic peaks results in more open chromatin for transcription factor binding, up‐regulating the expression of certain transcription factors via a feedback loop and ultimately increasing the expression of several developmental, metabolic, and hormone signaling pathways in the lateral root apical region. This shift ultimately modulates the RSA to predominantly increase lateral root growth to better mine phosphorus from low P soils. [file PLD3-6-e393-s008.pdf]

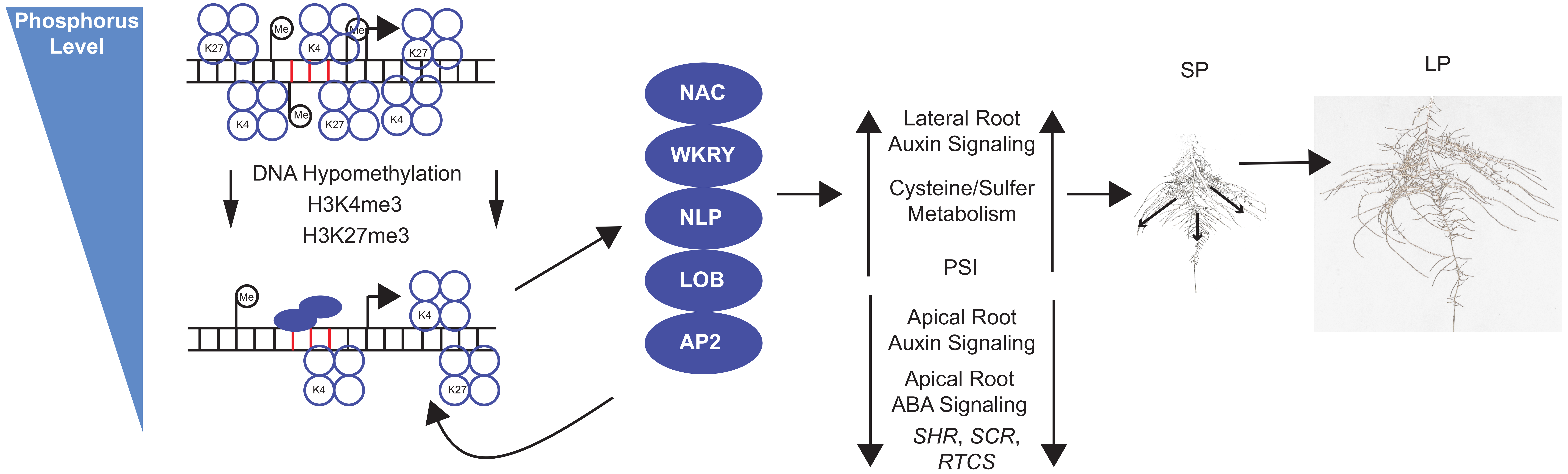

Phosphorus Level
K27
Me
K4
Me
K27
K4
Me
K27
K4
DNA Hypomethylation
H3K4me3
H3K27me3
Me
K4
K4
K27
NAC
WKRY
NLP
LOB
AP2
Lateral Root
Auxin Signaling
Cysteine/Sulfer
Metabolism
PSI
Apical Root
Auxin Signaling
Apical Root
ABA Signaling
SHR, *SCR*,
RTCS
SP
LP
